# Case Report: Genotype-informed management after clinically necessary cytoreduction for giant recurrent gastric gastrointestinal stromal tumor with prior rupture and KIT exon 17 N822Y mutation

**DOI:** 10.3389/fonc.2026.1908617

**Published:** 2026-10-08

**Authors:** Jianrui Rui, Chaoqiang Cai, Yongyuan Chen, Tianbao Xu, Zhen Wang, Guanxiang Zhuo, Linhe Gan, Liting Xue, Xueying Zeng, Min Zhou, Weiji Ding

**Affiliations:** 1 Department of Abdominal Surgery, Xiamen Cancer Hospital, The First Affiliated Hospital of Xiamen University, Xiamen, China; 2 Department of Radiology, Xiamen Cancer Hospital, The First Affiliated Hospital of Xiamen University, Xiamen, China; 3 Department of Pathology, Xiamen Cancer Hospital, The First Affiliated Hospital of Xiamen University, Xiamen, China

**Keywords:** case report, cytoreductive surgery, gastrointestinal stromal tumor, imatinib adherence, KIT exon 17, next-generation sequencing, ripretinib, tumor rupture

## Abstract

Advanced recurrent gastrointestinal stromal tumor (GIST) after prolonged tyrosine kinase inhibitor (TKI) exposure requires integration of surgical oncology, molecular reassessment, and continuous systemic therapy. We report a 72-year-old man with a 21-year history of gastric GIST who underwent initial resection in 2005, resection of recurrent disease in 2009, and long-term imatinib therapy. After years of apparent disease stability, he independently reduced imatinib and had a prolonged surveillance gap before recurrence was detected in 2024. Following tumor rupture and short-interval progression on sunitinib and regorafenib, he received ripretinib. In January 2026, clinically necessary maximal cytoreductive surgery was performed for bulky recurrent peritoneal disease with bleeding, anemia, abdominal-wall involvement, high risk of re-rupture, and invasion or close involvement of the right colon. Pathology confirmed recurrent high-risk spindle-cell GIST. Postoperative next-generation sequencing identified a founding KIT exon 11 deletion and a secondary KIT exon 17 p.Asn822Tyr activation-loop mutation; ripretinib was resumed after recovery of oral intake. Three-month CT showed recurrent peritoneal tumors, while a right hepatic subcapsular low-enhancement nodule was favored to represent post-ablation change. This case illustrates that surgery in resistant recurrent GIST may be necessary for local complication control and tissue acquisition, but even after maximal macroscopic cytoreduction, durable management depends on continuous genotype-informed systemic therapy and close surveillance.

## Introduction

Gastrointestinal stromal tumors (GISTs) are driven in most patients by oncogenic KIT or PDGFRA alterations, and management of advanced disease requires mutation testing, tyrosine kinase inhibitor (TKI) therapy, and multidisciplinary decision-making (1, 2). Tumor rupture may release viable tumor cells into the peritoneal cavity, resulting in intraperitoneal contamination and a high risk of subsequent peritoneal recurrence even after macroscopic resection (3–5). Long-term control on imatinib also does not equal cure in recurrent or metastatic GIST; BFR14 and related data support continuous imatinib suppression in non-progressive advanced disease rather than unsupervised discontinuation or dose reduction (6–8). The surgical role in advanced GIST is therefore nuanced. Surgery cannot replace systemic treatment, particularly after acquired kinase-domain resistance, but may become unavoidable because of bleeding, impending rupture, obstruction, bulky symptomatic disease, bowel invasion, or the need for tissue acquisition for molecular reassessment. We report a surgically and molecularly complex case of giant recurrent gastric GIST with prior rupture, in which a surveillance gap, short-lived later-line TKI control, clinically necessary cytoreduction, postoperative ripretinib resumption, and a secondary KIT exon 17 N822Y mutation converged in one patient.

## Case description

A 72-year-old man with no relevant family history underwent resection of a primary gastric GIST in Hong Kong in 2005; adjuvant therapy was not administered. In 2009, recurrence involving the small bowel and omentum was detected. Imatinib was initiated at 400 mg/day and temporarily escalated to 800 mg/day before metastatic deposits were resected at Fudan University Shanghai Cancer Center. Long-term imatinib 400 mg/day was subsequently maintained. In 2019, after many years without evident recurrence, the patient independently reduced imatinib to 200 mg/day because he interpreted prolonged disease stability as indicating that full-dose treatment was no longer necessary. He did not undergo regular CT surveillance between 2019 and 2024. This chronology is clinically relevant because patient-directed dose reduction coincided with a prolonged surveillance gap, which may have delayed recognition of recurrent disease. However, this single case cannot establish a causal relationship between dose reduction and subsequent recurrence. The complete clinical course is summarized in **Table 1**. Recurrent abdominal disease was identified in 2024. Imatinib was restarted according to the available history, but spontaneous tumor rupture occurred in November 2024 and required emergency peritoneal surgery at an outside institution. After this emergency surgery, progressive peritoneal disease was treated sequentially with standard-dose sunitinib as second-line therapy and standard-dose regorafenib as third-line therapy. Local CT after approximately one month of sunitinib showed tumor enlargement, and PET-CT after approximately one month of regorafenib showed continued enlargement; the regimen was therefore changed to ripretinib. Despite later-line treatment, abdominal disease progressed and the patient was admitted to Xiamen Cancer Hospital, formerly known as Fudan University Shanghai Cancer Center Xiamen Hospital, in January 2026 with progressive abdominal distension, melena, fatigue, reduced appetite, anemia requiring stabilization, and a firm palpable abdominal mass measuring approximately 15 × 15 cm. His Eastern Cooperative Oncology Group performance status was 1.

**Table 1:** Clinical timeline from initial gastric GIST resection to postoperative molecularly guided management.

Date	Clinical event	Management/outcome
2005	Primary gastric GIST resection in Hong Kong	No adjuvant therapy
2009	Recurrent small-bowel and omental disease	Imatinib 400 mg/day, temporarily escalated to 800 mg/day; metastasectomy; long-term imatinib 400 mg/day thereafter
2019	Patient-directed imatinib dose reduction	Patient reduced imatinib to 200 mg/day because he believed long disease control meant full-dose therapy was no longer needed
2019-2024	Surveillance gap	No regular CT surveillance according to patient history
Jun 2024	CT-confirmed recurrent abdominal disease	Imatinib restarted/adjusted according to available history; detailed imaging timeline incomplete
Nov 2024	Spontaneous tumor rupture	Emergency peritoneal tumor resection at an outside hospital. The original outside molecular report, subsequently retrieved for verification, documented a KIT exon 17 D816E mutation (c.2448C>A; p.D816E).
2025	Progressive peritoneal disease despite later-line TKIs	Sunitinib (~1 month): CT progression. Regorafenib (~1 month): PET-CT progression. Switched to ripretinib
Jan 22, 2026	Admission with abdominal distension, melena, fatigue, anemia, and 15 × 15 cm palpable mass	Stabilization; surgery judged clinically necessary because of giant symptomatic disease, bleeding, rupture risk, and bowel involvement
Jan 27, 2026	High-complexity exploratory laparotomy	434-min laparotomy; extensive adhesiolysis; maximal safe clearance of isolated visible nodules; tumor resection, right hemicolectomy, hepatic-edge microwave ablation, abdominal-wall repair
Feb 2, 2026	Final pathology	High-risk spindle-cell GIST; dominant 18 × 18 × 14 cm mass; mitotic count 20/5 mm²; Ki-67 ~50%; peritoneal/abdominal-wall dissemination; 0/15 nodes
Feb 12, 2026	Postoperative NGS report	KIT exon 11 AF 71.88%; KIT exon 17 N822Y AF 22.21%; MSS; no key fusion/CNV
Postoperative course	Uneventful postoperative recovery	No postoperative complications occurred; oral intake recovered, and ripretinib was subsequently resumed.
Apr 22, 2026	Three-month surveillance CT	New/enlarged peritoneal nodules up to 23 × 18 mm; right hepatic subcapsular low-enhancement nodule favored to represent post-ablation change

AF, allele frequency; CNV, copy-number variation; GIST, gastrointestinal stromal tumor; MSS, microsatellite stable; NGS, next-generation sequencing; TKI, tyrosine kinase inhibitor.

## Diagnostic assessment

Preoperative contrast-enhanced CT demonstrated multiple heterogeneous peritoneal masses with necrotic components, with the largest lesion measuring approximately 15 cm; two dominant recurrent abdominal tumors are shown in **Figure 1**. No definite solid parenchymal hepatic metastasis was identified. Given the patient’s history of tumor rupture in 2024, gastrointestinal bleeding, anemia, bulky recurrent peritoneal disease, threatened re-rupture, and threatened or established bowel involvement, the clinical context had moved beyond elective debulking. These findings created a practical threshold for clinically necessary surgery aimed at local complication control, macroscopic tumor-burden reduction, tissue acquisition, and preservation of postoperative systemic treatment feasibility.

**Figure 1:**
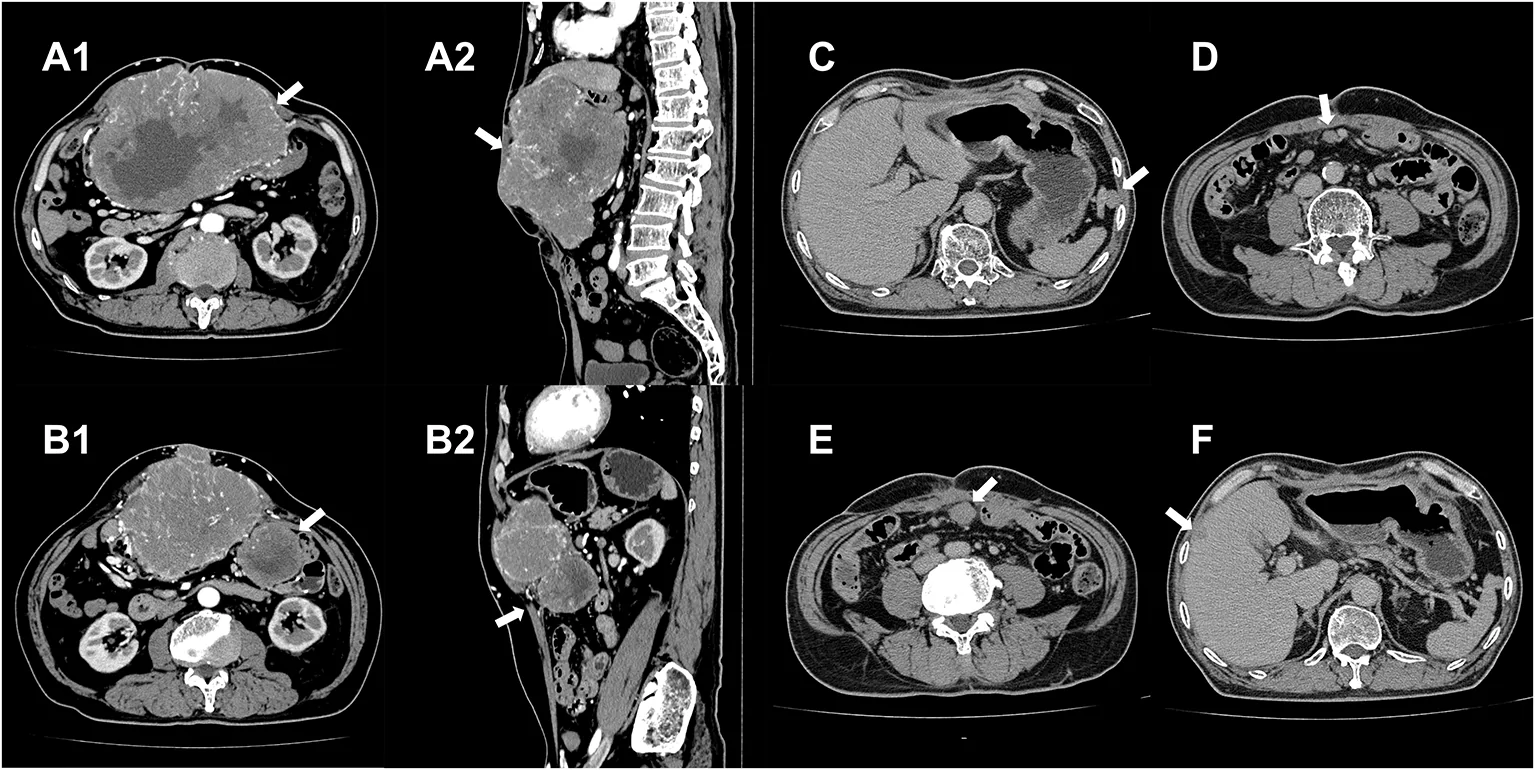
Representative preoperative and three-month postoperative CT findings. **(A1, A2)** Preoperative contrast-enhanced CT images showing the first dominant recurrent abdominal tumor in axial and sagittal planes. **(B1, B2)** Preoperative contrast-enhanced CT images showing the second dominant recurrent abdominal tumor in axial and sagittal planes. **(C–E)** Axial contrast-enhanced CT images showing selected recurrent peritoneal nodules/tumors at the three-month postoperative follow-up. **(F)** Axial contrast-enhanced CT image showing a right hepatic subcapsular low-enhancement nodule, favored to represent post-ablation change. Arrows indicate the lesions of interest.

Postoperative pathology confirmed recurrent high-risk spindle-cell GIST. The dominant tumor measured 18 × 18 × 14 cm, and additional peritoneal and abdominal-wall deposits were identified. Mitotic count was 20/5 mm^2^, necrosis involved approximately 10% of tumor volume, and Ki-67 was approximately 50%. Immunohistochemistry showed diffuse, strong cytoplasmic and/or membranous staining for CD117 and DOG1 in tumor cells. Tumor cells were negative for CD34, whereas vascular endothelial cells showed positive staining, serving as an internal positive control for CD34. Tumor cells showed diffuse, strong cytoplasmic SDHB staining, consistent with retained expression. The right hemicolon specimen showed focal tumor involvement of the muscularis propria; both bowel margins were negative, and 0/15 lymph nodes contained tumor. The tumor was classified as high risk by the modified NIH 2008 scheme. Representative gross and histological findings are shown in **Figure 2**. Postoperative next-generation sequencing (NGS) was performed on formalin-fixed paraffin-embedded tumor tissue using a 201-gene panel. Tumor cell content was approximately 90%, and sequencing quality metrics were acceptable. The assay detected a primary KIT exon 11 in-frame deletion, c.1674_1724del (p.Val559_Gln575del), with an allele frequency of 71.88%, and a secondary KIT exon 17 missense mutation, c.2464A>T (p.Asn822Tyr), with an allele frequency of 22.21%. The tumor was microsatellite stable, and no clinically significant gene fusion or copy-number alteration was reported. Variants of uncertain significance were not considered actionable. The original outside molecular report from 2024 was retrieved and documented a KIT exon 17 D816E alteration (c.2448C>A; p.D816E). This differed from the KIT exon 17 N822Y finding reported in the 2026 surgical specimen. To address this discrepancy, the original sequencing data were reanalyzed by our institutional laboratory, and the reported N822Y call was retained.

**Figure 2:**
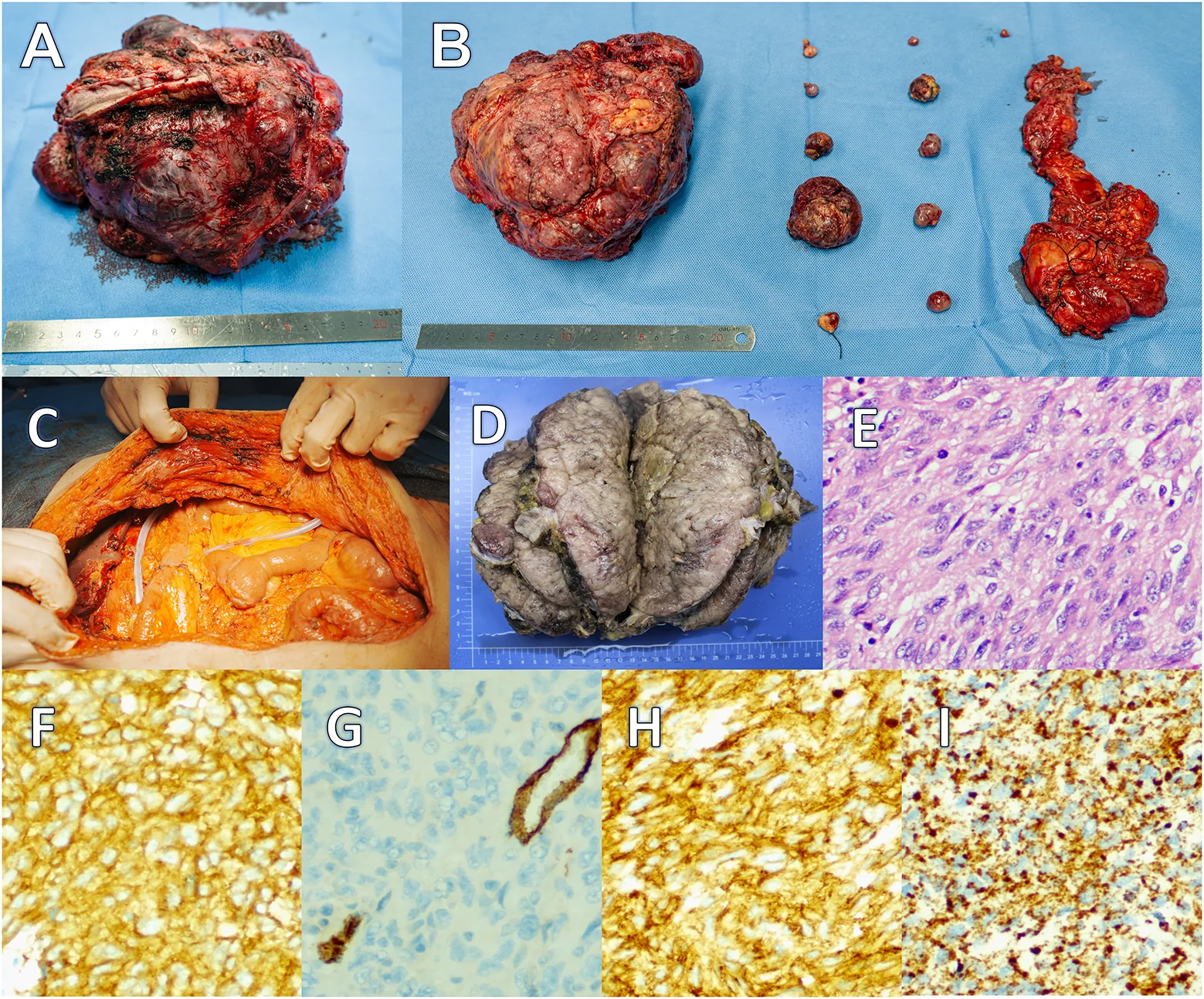
Gross specimens, intraoperative findings, histopathology, and immunohistochemistry of the resected recurrent gastrointestinal stromal tumor. **(A)** Anterior view of the resected surgical specimen showing the external appearance of the tumor. **(B)** Gross photograph of all resected specimens, including multiple tumor nodules of varying sizes and a segment of colon involved by tumor. **(C)** Intraoperative view of the operative field after tumor resection and before abdominal closure. **(D)** Cut surface of the resected specimen showing a solid tan-white tumor with a heterogeneous appearance. **(E)** Hematoxylin and eosin staining showing a hypercellular spindle-cell neoplasm arranged in interlacing fascicles, consistent with recurrent spindle-cell gastrointestinal stromal tumor. **(F)** Diffuse, strong cytoplasmic and/or membranous CD117 staining in tumor cells. **(G)** Negative CD34 staining in tumor cells, with positive staining in vascular endothelial cells serving as an internal positive control. **(H)** Diffuse, strong cytoplasmic and/or membranous DOG1 staining in tumor cells. **(I)** Diffuse, strong cytoplasmic SDHB staining in tumor cells, indicating retained expression. Original magnification: ×200 for **(E–I)**.

Further verification of the 2024 outside result could not be completed because of logistical barriers to accessing the outside material, including geographical distance, time requirements, and associated costs. Verification of the 2026 finding does not resolve the discrepancy between the two reports. Biological heterogeneity or temporal evolution, analytical or reporting error, and specimen-identity issues remain possible explanations. The available evidence does not establish when N822Y emerged or demonstrate a clonal transition from D816E to N822Y. Clinically relevant postoperative molecular findings are summarized in **Table 2**.

**Table 2:** Key postoperative next-generation sequencing findings.

Gene/status	Nucleotide/amino-acid change	AF	Class	Clinical interpretation
MSS status	55/55 microsatellite loci evaluable; microsatellite stable	N/A	Negative	MSI-H-directed checkpoint inhibition not supported by this result
KIT exon 11	c.1674_1724del (p.Val559_Gln575del)	71.88%	Clinically significant/pathogenic	Founding driver; compatible with primary imatinib-sensitive genotype
KIT exon 17	c.2464A>T (p.Asn822Tyr; N822Y)	22.21%	Clinically significant/potentially pathogenic	Secondary activation-loop resistance mutation; relevant to later-line TKI selection
VUS	PTCH2 p.Ala436Val; TET2 p.His658Tyr; FAT1 p.Glu1141Asp; NF1 p.Glu785Lys; HOXB13 p.Ala38Glu	Multiple	VUS	Not actionable for GIST management
Fusions/CNV	No key gene fusion or copy-number alteration detected	N/A	Negative	No additional actionable fusion or amplification identified

AF, allele frequency; CNV, copy-number variation; MSS, microsatellite stable; MSI-H, microsatellite instability-high; TKI, tyrosine kinase inhibitor; VUS, variant of uncertain significance.

### Therapeutic intervention

On January 27, 2026, exploratory laparotomy was performed. Extensive adhesiolysis was required because of severe intra-abdominal adhesions related to multiple previous abdominal operations, after which the abdominal cavity was systematically explored. Multiple isolated visible peritoneal and abdominal-wall nodules were removed when a safe dissection plane could be established and resection could be completed without a disproportionate risk of major vascular injury, unintended bowel injury, or excessive loss of functional bowel. The procedure included maximal macroscopic resection of visible peritoneal disease, resection of the dominant mass, right hemicolectomy because of tumor invasion and close involvement of the right colon, abdominal-wall repair, and microwave ablation of hepatic-edge lesions. Operative time, defined as the interval from skin incision to completion of skin closure, was 434 minutes. The procedure was technically demanding because adhesiolysis was time-consuming, the tumors were adherent to the abdominal wall and bowel, and visible-nodule clearance had to be balanced against the risks of intestinal and vascular injury. The operation is best described as clinically necessary maximal macroscopic cytoreduction rather than curative resection because microscopic residual disease and early recurrent peritoneal disease could not be excluded in this heavily pretreated patient with prior tumor rupture.

#### Follow-up and outcomes

The postoperative course was uneventful, and no postoperative complications occurred. Oral intake subsequently recovered, after which ripretinib was resumed. Three-month CT demonstrated recurrent peritoneal tumors in the pelvis, lower abdomen, and upper abdomen. A low-enhancement focus beneath the right hepatic capsule was noted near the region of prior hepatic-edge ablation; in light of intraoperative ablation, this focus was favored to represent post-ablation change rather than definite hepatic metastasis. Chest CT showed no thoracic metastatic disease. Representative preoperative and three-month postoperative CT findings are shown together in **Figure 1**.

## Discussion

This case has several messages relevant to oncology and surgical oncology practice. First, durable apparent control on imatinib should not be equated with cure in recurrent or metastatic GIST. The patient reduced imatinib without medical guidance and then had a prolonged surveillance gap. The later recurrence cannot be attributed solely to dose reduction in a single case, but the sequence is biologically plausible and clinically educational. Although BFR14 primarily evaluated imatinib interruption rather than dose reduction, its long-term follow-up supports the broader clinical principle that durable disease control in advanced GIST depends on sustained TKI suppression and careful surveillance; a Korean series of patients who interrupted imatinib after prolonged maintenance without gross disease similarly showed progression in most patients (6–8). This experience also underscores the need for repeated counseling that long-term TKI therapy in advanced GIST is suppressive rather than curative, confirmation of patient understanding, and structured medication-adherence and imaging-surveillance planning.Second, tumor rupture reframes prognosis. Ruptured GIST is associated with peritoneal relapse and adverse outcome and should be treated as a major biological turning point rather than only a mechanical complication (3–5). In this patient, rupture occurred in a tumor that had already demonstrated late recurrence after long-term TKI exposure. Even a technically thorough operation could not eliminate the risk created by prior intraperitoneal contamination, resistant subclones, and disseminated microscopic disease.

Third, the surgical indication should be framed accurately within the treatment sequence. After disease progression during long-term imatinib exposure, recurrent abdominal disease was detected in 2024, imatinib was restarted, and tumor rupture subsequently occurred, requiring emergency surgery at a local hospital. Postoperative sunitinib and regorafenib did not achieve meaningful disease control, and treatment was subsequently switched to ripretinib. The later operation at our center was not performed because cytoreduction was expected to overcome TKI-resistant GIST, but because bulky recurrent peritoneal disease, gastrointestinal bleeding, anemia, high risk of re-rupture, and tumor invasion or close involvement of the right colon created a practical threshold for clinically necessary surgery. Thus, the operation is best interpreted as local complication control, macroscopic tumor-burden reduction, and tissue acquisition to guide further systemic therapy. Sunitinib and regorafenib remain established post-imatinib agents in advanced GIST, but failure to achieve disease control with these agents in this patient reinforced the need for postoperative molecular reassessment and genotype-informed systemic treatment (9, 10). The operation reduced gross disease, controlled local complications, provided high-quality tissue for NGS, and allowed ripretinib to be resumed after oral intake recovered. Retrospective and multi-institutional data suggest that cytoreduction may help selected patients with metastatic GIST, especially when disease is controlled or complications drive the indication and surgery is combined with ongoing TKI therapy, but patient selection and systemic disease biology remain decisive (11–13).The molecular results explain why postoperative management could not rely on surgery alone. The high-frequency KIT exon 11 deletion was consistent with the founding imatinib-sensitive clone, whereas the lower-frequency KIT exon 17 N822Y mutation was consistent with a secondary activation-loop resistance alteration. Secondary activation-loop mutations are key mechanisms of acquired TKI resistance and may show differential sensitivity across agents (14–16). However, these general observations do not establish the timing of N822Y emergence, a D816E-to-N822Y clonal transition, or mutation-specific treatment benefit in this patient. Ripretinib had already been initiated before surgery, following treatment with imatinib, sunitinib, and regorafenib, and was resumed after recovery of oral intake. The postoperative NGS result therefore did not determine the initial selection of ripretinib. INVICTUS supports ripretinib in later-line advanced GIST, while INTRIGUE did not show overall superiority over sunitinib in the unselected second-line population; however, its ctDNA analysis suggested that patients with KIT exon 11 plus exon 17/18 secondary mutations may derive greater benefit from ripretinib than from sunitinib, a strategy now being prospectively tested in INSIGHT (14, 17–19).Three-month CT demonstrated recurrent peritoneal disease despite maximal macroscopic cytoreduction and postoperative ripretinib resumption. This does not negate the surgical indication; rather, it emphasizes that surgery in this setting is a bridge to systemic control, not an alternative to it. Despite radiologic evidence of recurrent peritoneal disease, the patient reported marked relief of abdominal distension, improved appetite and daily functioning, and an overall improvement in quality of life compared with his preoperative condition. This patient-reported benefit supports the palliative and local complication-control value of surgery without implying durable oncological control. The right hepatic subcapsular focus should be interpreted cautiously because its location near the ablation region favors a post-ablation scar or necrotic change rather than definite liver metastasis. This report is limited by its single-patient design, limited access to archival material from earlier operations, incomplete dates for later-line TKI treatment, and short oncological follow-up. A major unresolved limitation is the discordance between the KIT exon 17 D816E alteration documented in the outside report from 2024 and the N822Y finding in the 2026 surgical specimen. Although the latter was supported by verification in our institutional laboratory, further verification of the outside result could not be completed because of logistical and financial constraints. The present investigation therefore cannot fully distinguish biological heterogeneity or temporal evolution from analytical, reporting, or specimen-identity errors. Neither a D816E-to-N822Y clonal transition nor the timing of N822Y acquisition can be established. The molecular findings are reported transparently but are not used to claim mutation-specific therapeutic benefit. Future studies incorporating paired-specimen verification and longer follow-up are needed to clarify these questions.

## Conclusion

In giant recurrent GIST with prior rupture, bowel involvement, and secondary KIT exon 17 activation-loop resistance, maximal cytoreductive surgery may be unavoidable for local complication control and tissue acquisition. Durable management, however, depends on continuous genotype-informed systemic therapy, postoperative molecular reassessment, early restoration of effective TKI treatment when feasible, and close imaging follow-up.

## Data Availability

The data supporting the findings of this case report are included within the article. Additional de-identified clinical information may be available from the corresponding authors upon reasonable request, subject to institutional approval and patient privacy restrictions.
